# A grassroots approach to peer support by the Danish Reproducibility Network

**DOI:** 10.1186/s13104-024-06912-7

**Published:** 2024-09-10

**Authors:** Marta Topor, Philippe Bonnet, Veronika Cheplygina, Vibeke Høyrup Dam, Lorna Wildgaard

**Affiliations:** 1 Department of Behavioural Sciences and Learning, Linköping University, Linköping, Sweden; 2 Department of Nutrition, Exercise and Sports, University of Copenhagen, Copenhagen, Denmark; 3 Department of Computer Science, University of Copenhagen, Copenhagen, Denmark; 4 Department of Computer Science, IT University of Copenhagen, Copenhagen, Denmark; 5 Neurobiology Research Unit, Copenhagen University Hospital Rigshospitalet, Copenhagen, Denmark; 6 Royal Library, Copenhagen University Library, Copenhagen, Denmark

**Keywords:** Reproducibility network, Grassroots initiatives, Community building, Peer support, Reproducible research, Open research

## Abstract

The Danish Reproducibility Network (DKRN) is a grassroots initiative for establishing a peer-supportive reproducibility-focused academic network in Denmark. We modelled our approach on already existing national Reproducibility Networks. We consulted with researchers and research support professionals to identify the needs of the research community. Three themes emerged around policy implementation, training and the appropriate application of reproducible practices. The network aims to address these three themes in a strategic plan, which harnesses the benefits of grassroots initiatives. The mission of the DKRN is therefore to facilitate communication, peer-support, and the exchange of ideas through a network of topic and geographical nodes. The network is open to researchers and research support professionals from all career stages and disciplines. It aligns with broader international initiatives, and national institutions, positioning itself as a contributor to the Danish research ecosystem.

## Introduction

Complex and interconnected issues hindering research quality and integrity have been widely discussed in recent years. We often hear about the reproducibility crisis, which generally refers to the low success rate in reproducing published research findings [1, 2]. Closely connected is the increasing pressure on scholars to publish in high ranking journals to advance their career, possibly leading to inappropriate research practices [3, 4]. Problems with reproducibility are sustained by a lack of awareness and training among researchers and decision-makers responsible for research strategies and infrastructures at research institutions [5], even though guides on conducting responsible research have been readily available since before reproducibility became a “crisis”, for example p.8 in [6].

Science is believed to be self-correcting, however in the face of the reproducibility issues and given new developments for open and FAIR (findable, accessible, interoperable and reusable) science strategies, waiting for serendipitous corrections is not enough. All actors within the research ecosystem are now presented with the opportunity to coordinate efforts leading to better research quality, reproducibility and transparency [7].

As part of the coordinated action, many grassroots, peer-led networks have formed, for example, reproducibility networks (RNs) [8], open science communities [9] and journal clubs [10]. In this paper, we describe a case study of establishing the Danish Reproducibility Network (DKRN) inspired by the organisational structure first introduced by the UK Reproducibility Network [11]. Since the structure has now been adopted by more than 20 countries, our report may serve as a demonstration of how RNs can be used to address different needs of national research communities and inspire other emerging networks. Note that different RNs work in very different contexts with varying degrees of endorsement towards open and reproducible research by academic institutions and the relevant stakeholders. Our approach to establishing a network was informed by three steps:

1. Collecting views and perspectives on open and reproducible practices from Denmark-based research professionals and subsequently-
2. identifying the needs of the Denmark-based research community, which can be addressed through the DKRN’s activities.
3. Collecting information about the current national and institutional policies on open and reproducible research.

## Establishing a Reproducibility Network

The DKRN was registered as an association in March 2023 and joined the International Federation of Reproducibility Networks. The inaugural DKRN meeting was held on the 24th of August 2023 at the Royal Library, Copenhagen University Library^1^. We welcomed 44 in-person and 25 online attendees from 11 Danish research institutions as well as institutions outside of Denmark. 50% of the attendees were researchers with positions ranging from PhD student to full professor, and 50% were research support professionals including Data Protection Officers, Librarians, Data Stewards and IT and infrastructure developers. Majority of the attendees represented STEM, Table 1.

**Table 1:** Institutions and represented fields for attendees based in Denmark

Institution	Disciplines
Copenhagen Business School	Business
Danish Data Science Academy	STEM, SSH, Policy, Technology
Hvidovre Hospital	Neurobiology
IT University, Copenhagen	Digital Design, Systems and Applications, Computer science
Rigshospital	Neurobiology, Software Development,
Royal Library, Copenhagen	SSH, STEM, Policy
University Southern Denmark	Research Documentation
Technical University of Denmark	Infrastructure Development, Bio-sustainability, Technical and Natural Sciences
University of Copenhagen	Food and Resource Economics, STEM, Stem cell medicine, Experimental Medicine, Veterinary Science, Physics, Neuroscience
Aarhus University	Research Data Management, Urban Planning, Political Science, Psychology and Behavioural Science, Medicine, Pharmacology
Aalborg University	Data, Policy

During session one, representatives of national grassroots networks shared their experiences and advice. In session two, Denmark-based speakers discussed current strategies and projects for open and reproducible research. Attendees were encouraged to raise questions and share perspectives prior to and during the meeting. This was facilitated through specifically designed questions in the event sign-up form as well as shared brainstorming documents. All information was synthesised and discussed by the DKRN steering committee yielding the following themes:

- There is a need to improve communication and collaboration between research support services and researchers. For example, at the University of Copenhagen, the research support services promote strategies for the implementation of open research, FAIR data and data management policies. However, such new open science principles are evolving quicker than the academic culture itself. Researchers may not be interested in them, may have concerns about how to apply the principles or may not be aware of their importance.
- There is a need for information, methodological training and sharing of best practices for established and early-career researchers who are already aware of the shift towards improved research transparency and integrity.
- There is a need to carefully consider the relevance of different open and reproducible research practices for different fields. In his keynote at the inaugural meeting, Professor Jesper Schneider, Aarhus University, posed the question “Is reproducibility for all?”. He emphasised that open and reproducible research solutions are not necessarily suitable for all types of science and likewise scientific disciplines. As noted by the UK Reproducibility Network, one-size-fits-all approaches by institutions, funders and publishers will not work. There is a risk that open research practices will become a mere “box-ticking exercise” when mandated across diverse disciplines without the consideration of their practical application and relevance [4].

## Addressing community needs

The term “grassroots” reflects a bottom-up, community-driven initiative where researchers and research support professionals work together towards a common goal without an initial formal structure provided by institutions. Grassroot activities usually focus on the practical aspects of open and reproducible research including learning, developing and adopting new practices, identifying gaps in training or identifying barriers to implementation of new standards and policies [1]. DKRN as a grassroots network is very well placed to address the needs of the research community (see section above) as follows:

1. **Disseminate, discuss and provide peer-support on research standards and policies.** There are many recommendations, standards and policies released by global and national regulators as well as funders and publishers. For instance, in 2021 UNESCO published recommendations setting international standards for open science [12]. Major European research funders have or are in the process of implementing Plan S [13] to ensure open access publications for all funded projects. Publishers are widening open access in transformative agreements, increasing access to published research and removing or reducing APCs. Funders require researchers to openly share all research outputs including data, software and analysis code [14] to enable reproducibility.

In Denmark, the reproducibility crisis was highlighted by the Danish Ministry of Higher Education and Science in 2017 [15]. In 2018, the Ministry published a strategy for open access with a target set for 2025 as the year when all research institutions should publish only open access research articles [16]. By 2019, three major funders had already adopted an open access policy [17]. However, in a recent evaluation of the strategy’s implementation, the Ministry highlights the need for improved communication between the universities and the researchers regarding the open access strategies combined with clearer incentives [18]. As of today, six Danish universities and three funders have signed the CoARA agreement [19], which aims to encourage the provision of incentives for diverse outputs, practices and activities that maximise the quality and impact of research. Denmark also has a national strategy for FAIR data management [20], which contributes to increasing accessibility of publicly funded research. The remaining issue is that researchers are not always aware of these new policies or how to implement them in every-day practice. Through community building, the DKRN will help to raise awareness and facilitate peer-support relevant for a given institution or discipline. This objective directly corresponds to the first need of the Denmark-based research community as identified above.

2. **Help researchers navigate and discover relevant tools.** Researchers across the globe have been working to devise tools and solutions for improved research quality, transparency and integrity, which can elevate every single step within the research lifecycle [21]. These technological advances, however, may create significant barriers for researchers who are unfamiliar with them. The time needed to develop the skills to use the tools and solutions may lead to reluctance towards their adoption and the feeling of alienation in those who seem to be “falling behind”. A grassroots network can alleviate such issues in many ways. First, researchers will be encouraged to share specific reservations and concerns, which will in turn, identify training needs and barriers to the implementation of open and reproducible research practices at Danish institutions. Second, DKRN affiliates can support each other in navigating the relevant open and reproducible research tools and methodological solutions currently applied within their research fields, subsequently motivating discussions on efficient solutions within specific disciplines. Ideally, researchers and research support professionals in the DKRN can actively develop, adopt and/or adapt open and reproducible research practices together. This objective directly corresponds to the second and third need of the Denmark-based research community identified above.
3. **A potential for change on the institutional level.** Researchers who start adopting open and reproducible research practices can then share their experiences and knowledge with their immediate work environment. The visibility of open and reproducible research practices will increase and result in the wider adoption of new norms [9]. Subsequently, training needs for these norms will rise and this could motivate institutions in Denmark to design and offer discipline-specific training. In the broader perspective, advanced training on open and reproducible research methodology is important for the competitiveness of Denmark-based researchers on the international job market. As more institutions develop strategies for improved research integrity, hiring researchers with such skills may be prioritised. Conversely, researchers may prioritise universities with strong open and reproducible impact as places of employment or as research partners.

## Our vision for community and peer support

**Fig. 1:**
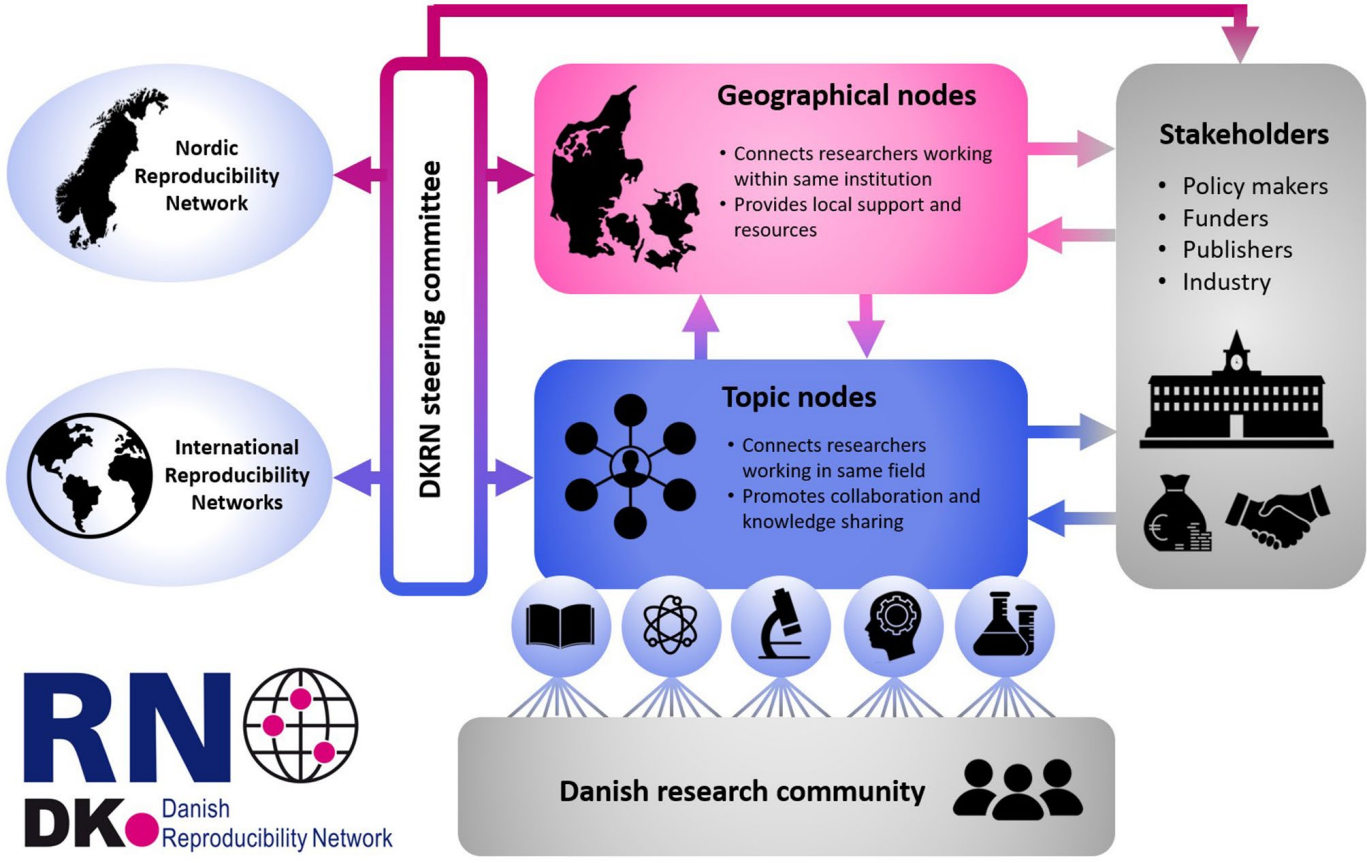
Overview of the proposed structure and coordination of the network

DKRNs envisioned structure is shown in Fig. 1. The core part of the network consists of geographical or topical community nodes. Geographical nodes are anchored to physical locations, e.g., academic institutions or towns, where they help connect individuals working in close proximity and provide local support and resources. Meanwhile, topical nodes are designed to create networks spanning the entire country by connecting individuals sharing specific scientific methods or interests across research institutions. The introduction of topical nodes is so far unique to the DKRN and was chosen to capitalise on the existing Danish research ecosystem, which includes a strong tradition for close collaboration between different research institutions. Denmark is a relatively small country and as such Danish research communities tend to be small and consequently better able to follow each other’s work. This combined with small geographical distances allows for frequent face-to-face scientific meetings and helps to foster national partnerships. The DKRN aims to be inclusive and collaborative where each topic and geographical node represents local and national resources that will help the research community to navigate the network according to their own interests and objectives. The node structure is appropriate for harnessing all benefits of a grassroots initiative described in Sect. 3. Specifically, the nodes will involve researchers and research support professionals who will bring knowledge and expertise on methods for open and reproducible research, methodological advancements and field standards, open science, data management, data stewardship, research ethics policies and other policies that impact how research is conducted, policy training and teaching materials. The structure emphasises the network’s priority focus on grassroots, bottom-up activity given the already existing and constantly developing top-down approaches.

The nodes are composed of at least one node lead and, if required, several contributors. The DKRN does not offer membership. There is no limit to how many nodes a person may be involved in. The network lead, contributors and the individuals involved in the activities of the network can be at any academic institution in Denmark, at any career level and of any academic profession (researcher, teacher, research support). We recognise that there are already many initiatives in Denmark, which aim to promote and enable the adoption of open and reproducible practices for the improvement of research integrity. Therefore, the nodes are not necessarily expected to organise new activities, but instead to collect information to disseminate across the network.

## Outlook

One leading cause preventing researchers’ engagement and contribution to RNs relates to the lack of incentives and recognition. The Knowledge Exchange collaboration, which involves many researchers and research support professionals based in Denmark, have worked with academic institutions to establish a framework for recognising and scaling up reproducible research [22]. The framework suggests different levels of recognition and dissemination of reproducible research practices as well as several enablers, which can help institutions to progress to the higher levels. These include, e.g. recognition in hiring and promotion or creating specific roles for expert advisers on open and reproducible research practices. It is the hope of the DKRN, that in parallel with our efforts, Danish research institutions will recognise the value of our activities and provide incentives for their researchers to contribute to our mission. Similarly, we hope that the principles of open and reproducible research will become recognised and incentivised by Danish research funders and national policymakers leading to an ideal scenario with synchronised efforts across both bottom-up and top-down levels.

## Data Availability

No datasets were generated or analysed during the current study.

## Footnotes

1 Materials available at https://danish-repro.github.io/.

## Abbreviations

APC: Article Processing Charges
CoARA: Coalition for Advancing Research Assessment
DKRN: The Danish Reproducibility Network
FAIR: The FAIR principles: Findability, Accessibility, Interoperability, and Reuse of research output
RNs: Reproducibility Networks
